# Combination of Two Kinds of Medicated Microparticles Based on Hyaluronic Acid or Chitosan for a Wound Healing Spray Patch

**DOI:** 10.3390/pharmaceutics13122195

**Published:** 2021-12-18

**Authors:** Angela Fabiano, Chiara Migone, Luca Cerri, Anna Maria Piras, Andrea Mezzetta, Giuseppantonio Maisetta, Semih Esin, Giovanna Batoni, Rossella Di Stefano, Ylenia Zambito

**Affiliations:** 1 Department of Pharmacy, University of Pisa, Via Bonanno 33, 56126 Pisa, Italy; angela.fabiano@unipi.it (A.F.); chiara.migone@for.unipi.it (C.M.); l.cerri3@student.unisi.it (L.C.); anna.piras@unipi.it (A.M.P.); andrea.mezzetta@unipi.it (A.M.); 2 Department of Life Sciences, University of Siena, Via Aldo Moro 2, 53100 Siena, Italy; 3 Department of Translational Research and New Technologies in Medicine and Surgery, University of Pisa, 56126 Pisa, Italy; giuseppantonio.maisetta@unipi.it (G.M.); semih.esin@med.unipi.it (S.E.); giovanna.batoni@med.unipi.it (G.B.); 4 Cardiovascular Research Laboratory, Department of Surgery, Medical, Molecular, and Critical Area Pathology, University of Pisa, Via Paradisa 2, 56100 Pisa, Italy; rossella.distefano@unipi.it; 5 Interdepartmental Research Centre “Nutraceuticals and Food for Health”, University of Pisa, 56100 Pisa, Italy

**Keywords:** olive leaves, hyaluronic acid, chitosan, microparticles, antibacterial activity, wound healing, spray patch

## Abstract

Olive leaves extract (OLE) has been extensively studied as antioxidant and antibiotic and these characteristics make it particularly interesting for use on wounds. For this reason, the aim of this study was to introduce OLE in microparticles (MP) of hyaluronic acid (MPHA-OLE) or chitosan (MPCs-OLE) to obtain a spray patch for the treatment of wounds in anatomical areas that are difficult to protect with traditional patches. The MP were characterized for particle size and ability to protect OLE from degradation, to absorb water from wound exudate, to control OLE release from MP. The MPHA and MPCs medicated or not and mixtures of the two types in different proportions were studied in vitro on fibroblasts by the scratch wound healing assay. The MP size was always less than 5 µm, and therefore, suitable for a spray patch. The MPCs-OLE could slow down the release of OLE therefore only about 60% of the polyphenols contained in it were released after 4 h. Both MPHA and MPCs could accelerate wound healing. A 50% MPHA-OLE-50% MPCs-OLE blend was the most suitable for accelerating wound healing. The MPHA-OLE-MPCs-OLE blends studied in this work were shown to have the characteristics suitable for a spray patch, thus giving a second life to the waste products of olive growers.

## 1. Introduction

Olive leaves are considered an agricultural by-product obtained during the harvesting or pruning process of olive fruits as they are burned by farmers resulting in the production of greenhouse gases [1]. For this reason, olive leaves conversion into higher value products can represent a sustainable and ecological alternative to leaves disposal. In this work, the leaves from *Olea europaea* cultivar *Olivastra seggianese* were considered. This tree has its origins in the Monte Amiata area (southern Tuscany, Italy), therefore its diffusion is limited to the provinces of Grosseto and Siena. These plants are interesting for their longevity, as they have survived the adverse climate of Monte Amiata and parasitic adversities over the centuries [2,3]. In fact, the olive leaf extract (OLE)—as well as olive oil—has been extensively studied for its antimicrobial and antioxidant characteristics, and also, as an antihypertensive, antiatherogenic, anti-inflammatory, hypoglycemic, or hypocholesterolemic agent [4,5,6,7,8,9]. These characteristics make this extract particularly interesting for use on wounds. For this reason, the aim of this study was to introduce OLE in microparticles (MP) of hyaluronic acid (HA) and in MP of chitosan (Cs) to obtain a spray patch for the treatment of wounds in anatomical areas difficult to protect with traditional patches, and also, in pets or farm animals.

HA is a widely used biomaterial in the field of wound dressings. Its properties during the wound healing process are related to its ability to bind to specific surface receptors CD44, RHAMM responsible for cell migration and proliferation. Cell migration is essential for the formation of a granulation tissue inherently rich in HA. In fact, the synthesis of HA is favored, generating a highly hydrated microenvironment [10] designed to stimulate the increase, proliferation, and migration of fibroblasts, endothelial cells, and keratinocytes [11]. Cs too has been extensively studied in wound healing [12]. It was found that Cs is able to promote the migration of macrophages, which lead to the phagocytosis of foreign bodies [13]. Furthermore, it has been shown that Cs in addition to activating macrophages, activates other inflammatory cells such as leukocytes, and fibroblasts, thus reducing the inflammatory phase and making the proliferative phase begin more rapidly [14,15].

However, it must be borne in mind that while Cs-based MP are capable of forming a transparent, compact, and resistant film on the wound [16], HA-based MP lack this ability [17]. Furthermore, only Cs possesses intrinsic antibacterial properties that could be added to those of OLE [18].

The microparticles of HA and Cs medicated with OLE could thus be sprayed on the wound and perform various actions, such as: (1) absorbing wound exudate thanks to the ability of the two polymers to absorb water; (2) promoting wound healing and protecting wounds against bacterial infections; (3) slowly releasing OLE in order to prolong the antibacterial effect of the device, thus avoiding frequent administration.

Spray patches based on mixed HA and Cs could be more effective in the treatment of wounds than those based on the single HA or Cs because the former could exploit the characteristics.

## 2. Materials and Methods

### 2.1. Materials

Olive leaves extract (OLE) preparation was carried out according to the previously reported procedure from *Olea europaea* var. *Olivastra seggianese cultivar* [19]. The leaves from which OLE was extracted were collected at CNR-IVALSA, Follonica (GR), Italy. Briefly, the freshly harvested olive leaves were freeze-dried (Freeze Dryer Modulyo, Edwards, Latina, Italy), then grounded in the presence of dry ice. The powder was dispersed in deionized water (1:5 *w*/*v*) and homogenized (IKA-ULTRA-TURRAX T25, Milan, Italy) for 5 min at 8000 rpm. Then the mixture was sonicated (Digital Ultrasonic Cleaner, Argolab 40 KHz, Milan, Italy) for 20 min at 35 °C, 40 KHz. The resulting suspension was centrifuged for 5 min at 4000 rpm and the supernatant was filtered through a 0.45 µm cellulose acetate filter. The filtrate was lyophilized (VirTis adVantage-53, Stereoglass, Perugia, Italy. Freezing −35 °C, 180 min; drying −30 °C, 360 min; −10 °C, 240 min; 25 °C, 180 min) and kept in the freezer until use.

Gallic acid and Folin–Ciocalteu reagent, as well as all the inorganic salts were purchased from Sigma (Milan, Italy). Hyaluronic acid (HA) was purchased from Contipro (Dolní Dobrouc, Czech Republic), had a molecular weight of 454.8 ± 103.6 kg/mol (determined by Debye plot method, zetasizer, Malvern). Commercial chitosan, minimum 90% deacetylated from shrimp shell (Chito-clear FG90, Primex, Drammen, Norway) and average viscometric molecular weight of 590 kDa [20] was converted into a chitosan·HCl powder (Cs). Cs was prepared by making an aqueous chitosan suspension (12 g in 2000 mL) to pH 4.7 with 1 N HCl (about 43.5 mL) and lyophilizing the resulting solution after filtration.

Murine embryonic fibroblasts BALB/3T3 clone A-31 cell line was purchased from the American Type Culture Collection LGC standards (ATCC HTB-37, Milan, Italy) and propagated as indicated by the supplier. Dulbecco’s modified Eagle medium (MEM), complete Dulbecco’s modified Eagle medium (DMEM), non-essential amino acid, 0.01 M pH 7.4 Dulbecco’s phosphate-buffered saline (DPBS), phosphate buffered-saline free of calcium and magnesium (PBSA), fetal bovine serum (FBS), and Hank’s balanced solution were purchased from Sigma (Milan, Italy). Cell proliferation reagent (WST-1) was provided by Roche diagnostic (Milan, Italy). *Staphylococcus aureus* 33591 and *Pseudomonas aeruginosa* 27853 were purchased from ATCC (Milan, Italy). Tryptone Soy Broth (TSB) and Tryptone Soy Agar (TSA) were purchased from Oxoid (Basingstoke, UK).

### 2.2. Total Polyphenol Content (TPC) Determination in Olive Leaves Water Extract (OLE)

The TPC in OLE was determined following the Folin–Ciocalteu colorimetric method as previously described [21]. Briefly, 1.58 mL of water and 100 µL of Folin–Ciocalteu reagent were added to a 50 µL aliquot of an OLE solution. After three min, 300 µL of sodium carbonate solution (1.9 M) was added and the absorbance was measured spectrophotometrically (Lambda 25 Perkin Elmer, Milan, Italy) at a wavelength of 765 nm after two hours of incubation in the dark.

The TPC was therefore determined by referring to the calibration curve obtained with gallic acid and expressed as mg per g of lyophilized OLE.

### 2.3. Bactericidal Assays

The reference strains *Staphylococcus aureus* and *Pseudomonas aeruginosa* were used in the present study. For liquid cultures, bacteria were grown in TSB at 37 °C with shaking. Enumeration of colony-forming units (CFU) was performed by serially diluting bacterial suspensions and plating them on TSA. CFU were counted after an incubation of 24 h at 37 °C.

The bactericidal activity of OLE was evaluated against *S. aureus* and *P. aeruginosa* in 5% TSB. To this aim, bacterial strains were grown in TSB until exponential phase, and a number of approximately 5 × 10^4^ CFU/mL, contained in a volume of 10 µL, were added to a mix solution composed by 50 µL of OLE (ranging from 2.5 to 20 mg/mL), 5 µL TSB, and 35 µL of water. The samples were incubated at 37 °C with shaking for 3 and 24 h. Following incubation, samples were 10-fold diluted in LB and plated on TSA to determine the number of CFU. Bactericidal activity was defined as a reduction of at least 3 Log10 in the number of viable bacteria as compared to the inoculum.

### 2.4. Preparation of Microparticles (MP) Using the Spray-Drying Technique

MP based on HA (MPHA) or Cs (MPCs) medicated with OLE (MPHA-OLE, MPCh-OLE), were prepared by spray-drying. A 0.5% *w*/*v* solution of HA or Cs in water was prepared and spray-dried. The medicated MP were prepared by adding OLE (10 mg/mL in deionized water) dropwise to the polymer solution under magnetic stirring in a polymer:OLE 10:1 wt ratio. The solutions obtained were nebulized using a spray-dryer (Büchi Mini Spray Dryer B-191, Cornaredo, Italy) setting the following conditions: feed flow 600/700 mL/min, aspirator 100%, pump flow rate 15%, air temperature 175–180 °C inlet, and 80 °C outlet temperature. To ensure the homogeneity of the nebulization, the solution was kept under continuous magnetic stirring.

### 2.5. MP Characterization

#### 2.5.1. Determination of the OLE Content in Medicated MP

To verify that the OLE content in the MPHA-OLE and MPCs-OLE corresponded to the nominal content (10 wt %), 10 mg of medicated MP were dissolved in 1 mL of deionized water. The solution was then analyzed for the TPC. To rule out any interference, the same procedure was applied to non-medicated MP.

#### 2.5.2. Dimensional and Morphological Analysis: Scanning Electron Microscopy (SEM)

The morphology (shape and surface characteristics) of the dried MP coated by metallization with 24 kt gold in a vacuum chamber at 15 mA for 20 min was observed by scanning electron microscope analysis (SEM, model JEOL JSM 300, Tokyo, Japan). ImageJ software was used to evaluate the MP average diameter. The images were divided into six grids with an area of 600 µm^2^. The mean diameter was calculated based on 40 measurements.

#### 2.5.3. Thermogravimetric Analysis (TGA)

The thermal stability of the MPHA-OLE, MPCs-OLE, and free OLE was investigated by using thermal gravimetric analysis (TA Instruments Q500 TGA, New Castle, DE, USA). The instrument was calibrated using weight standards (1 g and 100 mg) and the temperature was calibrated using nickel standard. All standards were supplied by TA Instruments Inc. The samples (10–15 mg) were heated at 40 °C in a platinum crucible for the drying procedure and maintained in N_2_ flux (90 mL/min) for 30 min. Then, the samples were heated from 40 °C to 600 °C with a heating rate of 10 °C/min under nitrogen (90 mL/min) and maintained at 600 °C for 3 min. Mass change was recorded as a function of temperature and time. TGA experiments were carried out in duplicate.

#### 2.5.4. Determination of OLE Stability in MP

The stability of the polyphenols in OLE was determined in wound simulating buffer (pseudo extracellular fluid PECF, 0.11 M NaCl; 0.03 M KCl; 0.03 M NaH_2_PO_4_ e 0.3 M NaHCO_3_, pH 8) [22]. Ninety mg of MPHA-OLE, MPCs-OLE, or free OLE was suspended in 2 mL of PECF and immersed in a thermostated bath at 37 °C temperature under continuous stirring. The final OLE concentration was 4.5 mg/mL. At 30 min intervals, for a total of 4 h, 50 μL were withdrawn, acidified with 1 M HCl, and analyzed for TPC quantification. These tests were performed in quadruplicate.

#### 2.5.5. Determination of the MP Swelling Degree

A previously reported technique was used to determine the MP swelling degree [23]. Briefly, 20 mg of each type of the MP under study were suspended in 2 mL of PECF pH 8 at 37 °C. After predetermined time intervals, the suspensions were filtered through paper filters. Each filter was then placed in a test tube and centrifuged at 2000 rpm for 2 min in order to remove excess water and weighed. To accurately evaluate the weight of the swollen MP, the same filters used for the tests were previously immersed in PECF, centrifuged at 2000 rpm for 2 min, and weighed and the weight of the swollen sample was calculated by difference.

The swelling degree was calculated according to the equation

Swelling degree (%) = [(Ws − Wfs)/(Wd − Wfd)] × 100(1)

where Wd and Wfd are the initial weights of dry sample and dry support filter, respectively. Ws and Wfs are the swollen weights of sample and support filter, respectively. The swelling degree at each time point was determined in triplicate.

#### 2.5.6. Study of the Release Profiles from Medicated MP

The OLE release from the medicated MP was determined by placing 100 mg of MPHA-OLE or MPCs-OLE in 5 mL of PECF pH 8 in a thermostated beaker at 37 °C, under stirring. At predetermined time intervals, samples of 50 µL were taken from the suspension and analyzed, after filtration (0.45 µm cellulose acetate filters), for TPC quantification.

### 2.6. Cell Culture Techniques

Biological evaluations of polymeric MP were conducted using the BALB/3T3 clone A31 murine embryonic fibroblast cell line.

#### 2.6.1. Cell Viability and Proliferation Test by WST-1 Assay

The cytotoxicity of MPHA, MPHA-OLE, MPCs, MPCs-OLE, and free OLE samples was evaluated by the WST-1 assay. The cells were seeded in 96-well plates, at a concentration of 7 × 10^3^ cells per well and allowed to proliferate for 24 h at 37 °C and 5% CO_2_. Stock dispersions of MP were prepared, previously made sterile by keeping them 20 min under UV, in DMEM without serum. Samples were diluted to obtain concentrations in the 0.5–50 µg/mL range of OLE, either free or loaded into MP, considering 1/10 wt of loading. After 24 h, the culture medium was removed and replaced with the samples, and left in contact with the cells for 4 h. At the end of the incubation time, the cells were incubated at 37 °C for 4 h with WST-1 diluted 1/10. The amount of formazan produced was evaluated by measuring the absorbance at 450 nm with a microplate reader. The cytotoxicity of MP mixtures composed of MPHA and MPCs, medicated and not, was also evaluated in the ratios 25:75, 50:50, and 75:25 (abbreviations: MPHA25MPCs75, MPHA50MPCs50, MPHA75MPCs25, MPHA25MPCs75-OLE, MPHA50MPCs50, MPHA75MPCs25-OLE) at the final MP concentration of 30 µg/mL.

#### 2.6.2. In Vitro Scratch Wound Healing Assay

The scratch wound healing assay is a method developed to assess in vitro the ability of cell migration and proliferation [24]. The cells were seeded, in 12-well plates, 1.25 × 10^5^ cells per well in DMEM medium containing 1% calf serum. Complete confluence was achieved after 24 h at 37 °C and 5% CO_2_. The resulting monolayer was scratched with a sterile pipette with p200 tip. After scratching, the wells of the plate were washed three times with PBSA to remove debris and various dead cells. Then 2 mL of each sample to be tested was added. MPHA, MPHA-OLE, MPCh, MPCh-OLE, and three mixtures composed of medicated and non-medicated MPHA and MPCh were analyzed (MPHA25MPCs75, MPHA50MPCs50, MPHA75MPCs25, MPHA25MPCs75-OLE, MPHA50MPCs50-OLE, and MPHA75MPCs25-OLE) at a concentration of 30 µg/mL, and the free OLE at a concentration of 3 µg/mL. In the control tests, the cells were incubated in the presence of the culture medium alone (DPBS).

Micrographic images of each well were acquired by means of a camera with a 4×-objective (Nikon Eclipse Ts2R) at time zero and after 24 h. The acquired images were analyzed using the ImageJ Software to determine the percentage of healing of the inflicted scratch.

The following formula was used to determine the percentage of healing over time

Wound healing (%) = [(Area T0 − Area T)/Area T0] × 100(2)

where Area T0 is the area at time zero, Area T is the area at 24 h.

## 3. Results

### 3.1. TPC Determination in OLE

OLE was characterized in previous studies [25,26]. The main components of this OLE are similar to those already found in OLE from other varieties of *Olea europaea* [27]. In this work, the same olive leaves were used from which the extract was previously obtained. For this reason, we only verified that the re-prepared extract had the same polyphenol content and the same antimicrobial activity as the extract previously obtained [25,26]. The content of total polyphenols (TPC) in the lyophilized OLE was in line with the previous one, resulting in 79.47 ± 6.99 mg per g of lyophilized OLE. Although reference is made to TPC, it should be kept in mind that the Folin–Ciocalteau reagent is not specific for phenolic compounds and could also react with other molecules present in the extract with reducing characteristics [28].

### 3.2. OLE Antibacterial Activity

**Figure 1:**
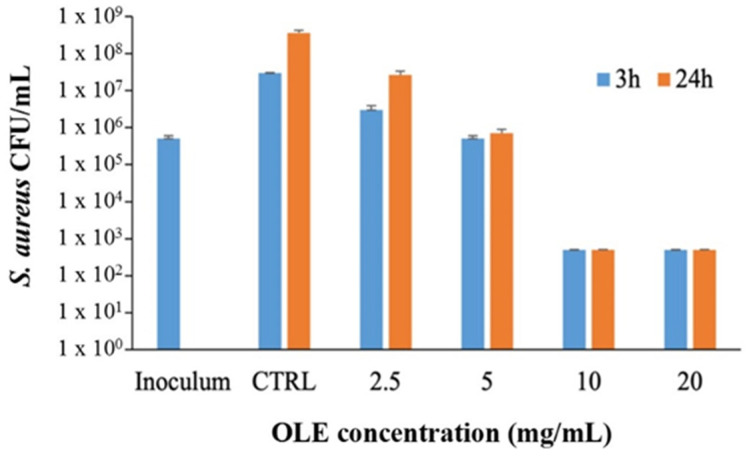
Antibacterial activity of OLE against *S. aureus* ATCC 33591 after 3 and 24 h of incubation in 5% TSB. Control (CTRL) represents bacteria incubated in the absence of OLE. Bactericidal activity was defined as a reduction in the numbers of viable bacteria of ≥3-log colony forming units (CFU)/mL at any incubation time tested. Data are reported as mean ± SD (*n* = 3).

In order to verify that the OLE used in this study had antimicrobial activity as already reported [26] was tested against *S. aureus* and *P. aeruginosa*, representative of Gram-positive and Gram-negative bacterial species respectively often involved in infection of wounds [29]. OLE, tested at 5 mg/mL, showed a bacteriostatic effect against *S. aureus* after both 3 and 24 h of incubation, Figure 1, whereas at 10 and 20 mg/mL OLE exerted a strong bactericidal effect, Figure 1. No antibacterial activity was observed when OLE was tested against *P. aeruginosa* until a concentration of 20 mg/mL (data not shown). These latter results are in agreement with previous studies that reported low susceptibility of *P. aeruginosa* to a number of plants derived oil extracts and in particular to olive oil extracts [30] and might be due to the intrinsic tolerability of this bacterial species to antimicrobials.

### 3.3. MP Characterization

**Figure 2:**
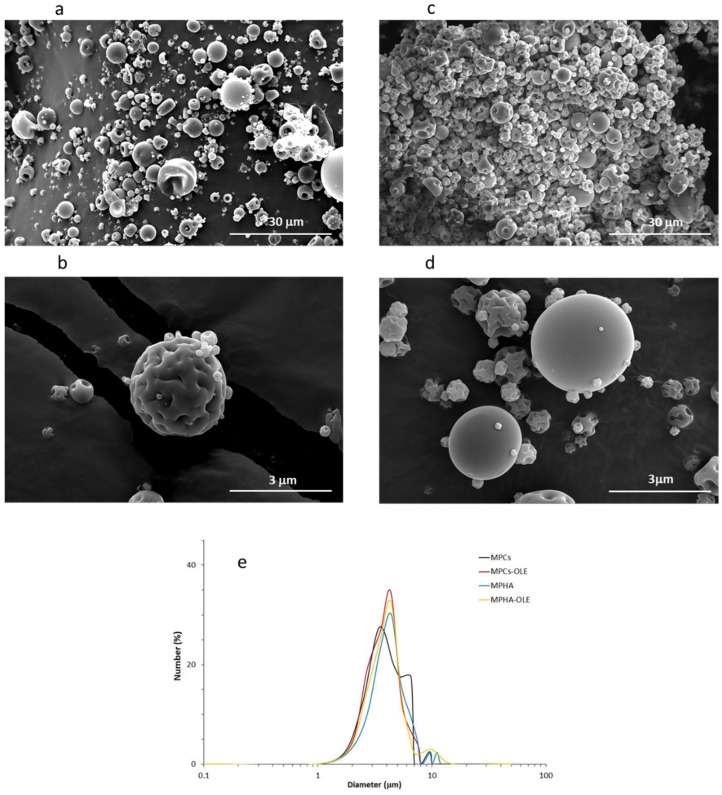
SEM micrographs of (**a**) MPHA; (**b**) MPHA-OLE; (**c**) MPCs; (**d**) MPCs-OLE; (**e**) Number (%) vs. diameter size distribution curves of MP.

Both MPHA-OLE and MPCs-OLE contained the nominal amount of OLE, i.e., 10% by weight. This result was indeed expected, since the spray-drying technique ensures that the active ingredient does not degrade during drying. Figure 2 shows the SEM micrographs of MPHA, MPCs, MPHA-OLE, and MPCs-OLE. In all cases, the majority of MP have a spherical shape, although some irregularly shaped MP can be observed. The average sizes of MPHA, MPHA-OLE, MPCs, and MPCs-OLE are 4.7 ± 1.8, 4.3 ± 1.3, 4.5 ± 1.6, and 4.0 ± 1.2 µm, respectively. This testifies to some dimensional dispersion, since, after all, smaller particles adsorbed on larger ones are observed in Figure 2. However, the average size is in all cases less than 5 µm and this makes the MP suitable for their topical administration [31]. It is also observed that the size of non-medicated MP does not differ significantly from medicated ones. Finally, it is noted that all kind of MP have a smooth surface even if in some cases they are collapsed. This is not surprising, rather, it suggests a hollow capsule morphology characteristic of the MP obtained by the spray-drying technique.

### 3.4. TGA Analysis

**Figure 3:**
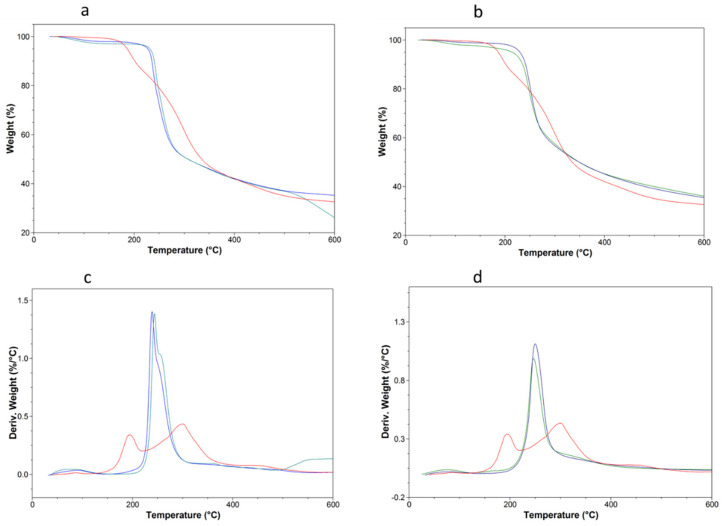
Thermogravimetric curves for (**a**) green MPHA, blue MPHA-OLE, red OLE; (**b**) green MPCs, blue MPCs-OLE, red OLE, and the respective derivative curves (**c**) and (**d**) (*n* = 2).

TGA analysis is a widely used technique to evaluate the thermal stability of compounds in which the loss of mass as a function of temperature is measured. Figure 3a,b show the thermogravimetric curves of MPHA, MPHA-OLE and MPCs, and MPCs-OLE, respectively, in comparison with free OLE. Figure 3c,d show the derivative of the respective thermogravimetric curves. Figure 3 shows that the weight loss of the samples decreases with increasing temperature. The degradation temperature (*T*_d_), which corresponds to the point of intersection between the starting-mass baseline and the tangent to the TGA curve at the point of maximum gradient reported in Figure 3, shows two degradation phenomena for OLE; the first around 170 °C and the second around 270 °C. The degradation curves of MPHA-OLE and MPCs-OLE are not significantly different from the curves of non-medicated MP. In the case of MPHA and MPHA-OLE (Figure 3c) a main degradation peak is observed at 230 °C with a shoulder at a higher temperature, indicating the overlapping of the breaking of the β(1–3) glycosidic bonds and the decomposition of carbonyl groups as reported in the literature [32]. In the case of MPCs and MPCs-OLE (Figure 3d), a degradation peak around 230 °C is observed, which could be attributed to the polysaccharide backbone depolymerization and pyrolytic decomposition of chitosan [33]. Despite the presence of OLE, its degradation profile is not revealed by the analysis of MPHA-OLE and MPCs-OLE particles. In particular, the first peak of OLE degradation at 170 °C totally disappears, suggesting an effective encapsulation of OLE in both MP types, with consequent protection of the extract from decomposition.

### 3.5. Determination of OLE Stability in MP

**Figure 4:**
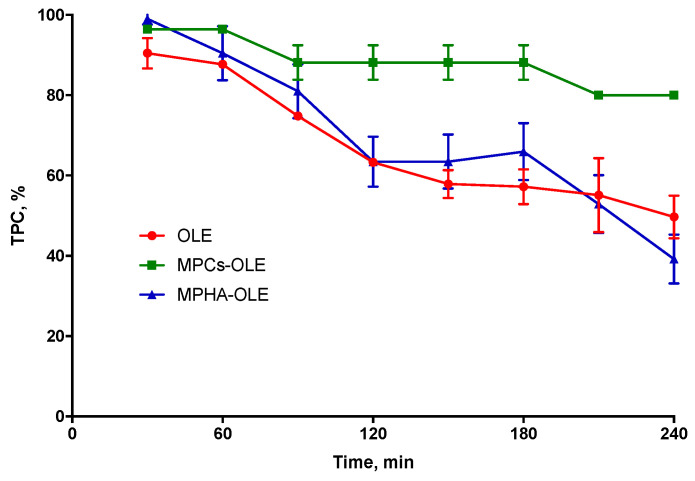
OLE stability in pseudo extracellular fluid (PECF) at 37 °C. Means ± SD (*n* = 4).

OLE stability in PECF medium was assessed by monitoring the variation of reactivity with the Folin–Ciocalteau reagent. Figure 4 shows the degradation curves of OLE in PECF medium over time, thus expressed as decreasing TPC % value. It is observed that the MPHA-OLE follows a degradation profile completely superimposable to that of non-encapsulated OLE showing their inability to slow down the degradation of OLE in solution. This could be due to a rapid release of OLE by the MPHA. Instead, in Figure 4 it is observed that after 4 h the MPCs-OLE are able to maintain the TPC at about 80% of the initial content. This testifies that chitosan microparticles are able to protect OLE from degradation even in the simulated wound exudate medium.

### 3.6. Determination the MP Swelling Degree

**Figure 5:**
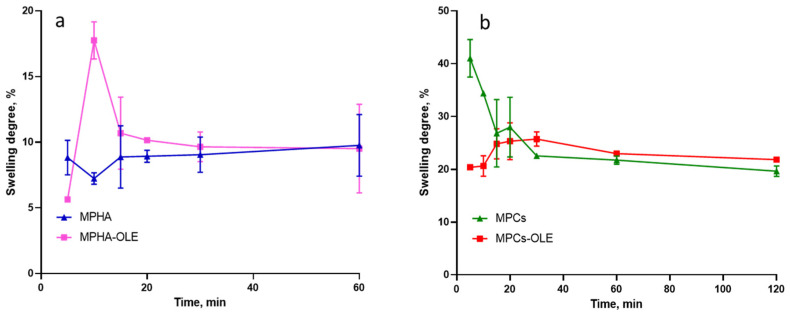
Swelling curves of (**a**) MPHA and MPHA-OLE and (**b**) MPCs and MPCs-OLE in pseudo extracellular fluid (PECF) at 37 °C. Means ± SD (*n* = 3).

The swelling profiles of medicated and non-medicated MPHA and MPCs are displayed in Figure 5. In Figure 5a it is observed that the MPHA swelling is very fast and reaches its equilibrium at the first time point (5 min). Furthermore, it retains the absorbed PECF for the entire experimental time (1 h). Differently, the MPHA-OLE show a swelling peak after 10 min, corresponding to a 50% increase of its initial weight. Evidently, the interaction between HA and OLE increased the osmotic force of the MP. In Figure 5b the MPCs show a maximum of absorption after 5 min. On the other hand, MPCs-OLE show a swelling peak after 30 min, with a swelling degree lower than that of non-medicated MPCs. Contrary to what observed with MPHA-OLE, the presence of OLE in the polymer matrix determines an interaction between the hydrocolloid and the polyphenols, which reduces the swelling behavior of MPCs-OLE [34]. These data suggest that all MP under study are particularly suitable for the preparation of a spray patch. In fact, a high hydration and swelling power is an essential property as it makes the formulation ideal for absorbing water from the exudate [16].

### 3.7. Study of Release Profile from Medicated MP

**Figure 6:**
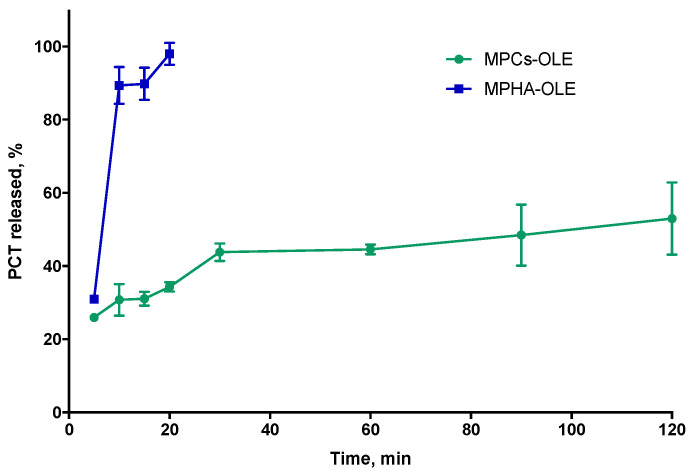
OLE release curves from MPHA-OLE or MPCs-OLE in pseudo extracellular fluid (PECF) at 37 °C. Means ± SD (*n* = 3).

To follow the release profiles of the active ingredients from the MP, the Folin–Ciocalteau analytical method was chosen, which allows identification of all the antioxidant species present in the solution. This methodology seemed more effective in this context precisely because most of the active molecules present in the extract—such as oleuropein, hydroxyl-tyrosol, apigenin-7-*O*-glucoside, luteolin-7-*O*-glucoside [26]—respond positively to the assay. Figure 6 shows the TPC percentage released over time by MPHA-OLE or MPCs-OLE in PECF, with respect to the TPC value of loaded OLE. From the data reported in Figure 6, it can be observed that in the case of MPHA-OLE the release is complete after 15 min. Moreover, in the present experimental conditions, in which there is a large excess of PECF, it is observed that the MPHA-OLE dissolve after forming a compact gel on the bottom of the container, which erodes quickly. The inability of HA to protect OLE from degradation, shown in Figure 4, may depend on OLE being completely released from the HA-based microparticles after just 15 min from the start of the release study. In the case of the MPCs-OLE the release was followed for a total time of 4 h. In fact, as seen in Figure 4, the OLE released after 4 h in PECF begins to degrade; therefore, the release kinetics at longer times could be distorted by the simultaneous degradation of the OLE already released. Contrary to what has been observed with MPHA-OLE, MPCs-OLE does not erode in the PECF alkaline buffer. In Figure 6, the release profile for MPCs-OLE shows that after 5 min about 25% of OLE is released. This may be due to a desorption of the OLE adsorbed on the external surface of the MP. In the subsequent 4 h, the release occurs slowly and reaches the value of about 60%. These data are in agreement with those previously obtained for a similar device [16]. The shape of the release curve, together with the swelling data shown in Figure 5a and the fact that the MPCs-OLE does not dissolve in the alkaline buffer, suggest that the release is controlled by the diffusion of the OLE from the MP.

### 3.8. Cell Viability and Proliferation Test by WST-1 Essay

MPHA, MPHA-OLE, MPCs, MPCs-OLE, and free OLE, were subjected to in vitro biological investigations for the evaluation of cytotoxicity. Cells from the BALB/3T3 clone A31 murine fibroblast line were incubated with MP for 4 h and subsequently analyzed by the WST-1 colorimetric assay. This assay was performed to select the best concentration to be used in subsequent wound healing studies.

**Figure 7:**
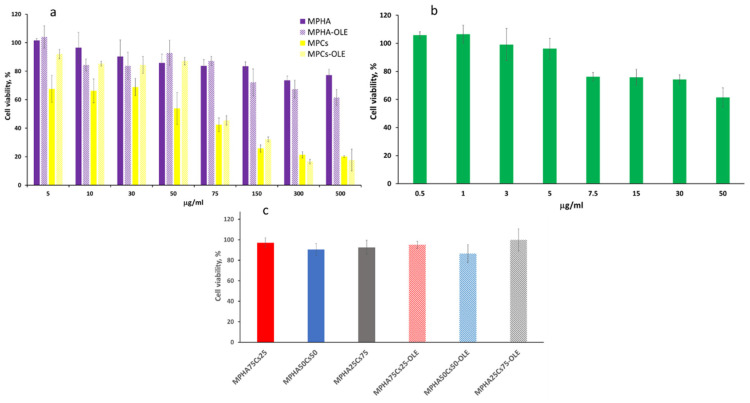
BALB/3T3 clone A31 murine embryonic fibroblast cell viability after 4 h of incubation with (**a**) MPHA, MPHA-OLE, MPCs, MPCs-OLE; (**b**) free OLE; (**c**) mixtures of MPHA and MPCs medicated or not (30 µg/mL total MP concentration). Data are expressed as % viable cells compared to 100% of control (untreated cells). Means ± SD (*n* = 8).

Neither MPHA nor MPHA-OLE showed cytotoxicity at any of the concentrations assayed, in the range 5–500 µg/mL. As shown in Figure 7a the cell viability value was always over 75% of the control cells viability (cells grown in the absence of MP), indicating a high biocompatibility. Instead, the MPCs and MPCs-OLE showed cell viability values around 60–80%, at the lowest concentration tested (5–50 µg/mL) while at higher concentrations (75–500 µg/mL) they showed values of cell viability around 20–40% (Figure 7a).

Free OLE also showed no cytotoxicity at the concentrations analyzed (0.5–50 µg/mL). However, it can be observed in Figure 7b that the percentage of cell viability decreases slowly with increasing concentration.

The results of the toxicity tests led to the choice of the maximum non-toxic MP concentration of 30 µg/mL, corresponding to 3 µg/mL of OLE, to be used in the subsequent wound healing tests. For this reason, the cytotoxicity of mixtures of HA and Cs based MP was also tested, by keeping constant the total MP concentration (30 µg/mL) while changing the MPHA/MPCs ratio (25/75, 50/50, or 75/25), either medicated or not. None of the mixtures tested were found to be toxic at the concentration chosen (Figure 7c).

### 3.9. In Vitro Scratch Wound Healing Assay

**Figure 8:**
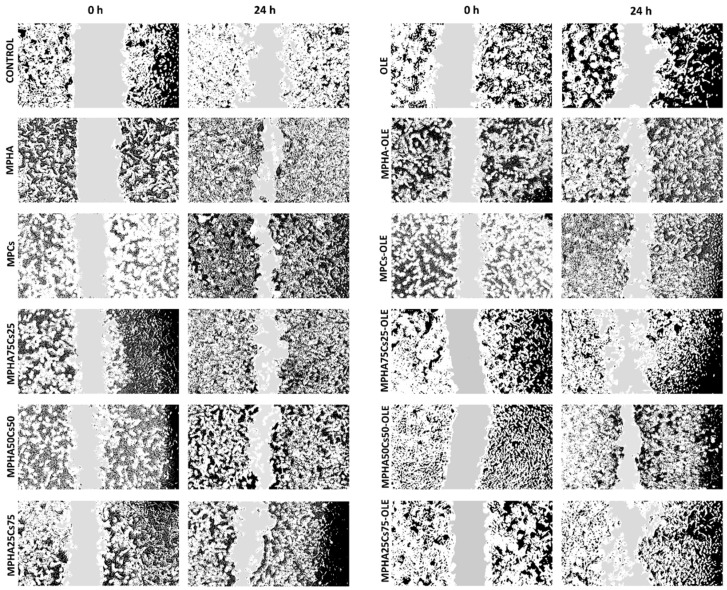
Representative micrographs (4× magnification) of BALB/3T3 clone A31 murine embryonic fibroblast cell monolayers, processed with ImmagJ software. CTRL, plain MPHA, MPCs, and their mixtures are displayed on the left side panel; OLE and medicated MP, as well as their mixtures are on the right side of the panel.

In the wound healing studies performed by scratch test, cell migration was evaluated in a two-dimensional manner on monolayer of Balb/3T3 cells, with respect to time. The in vitro scratch test is the first choice method for analyzing cell migration because it does not require any specialized equipment and all the necessary materials are available in any laboratory performing cell culture studies [35]. The technique involves the creation of a thin and linear ‘wound’ by scratching a monolayer of cells and subsequent acquisition of images of the cells that migrate towards the space created [36]. Low serum concentrations (1% FBS) in the cell medium were used to reduce cell proliferation and promote cell migration. Figure 8 displays a panel of micrographs, processed with ImageJ. From this first qualitative investigation it is possible to note that at time zero only the edges of the scratch are present and that after 24 h it is possible to observe the migration from the margin towards the center of the simulated wound.

**Figure 9:**
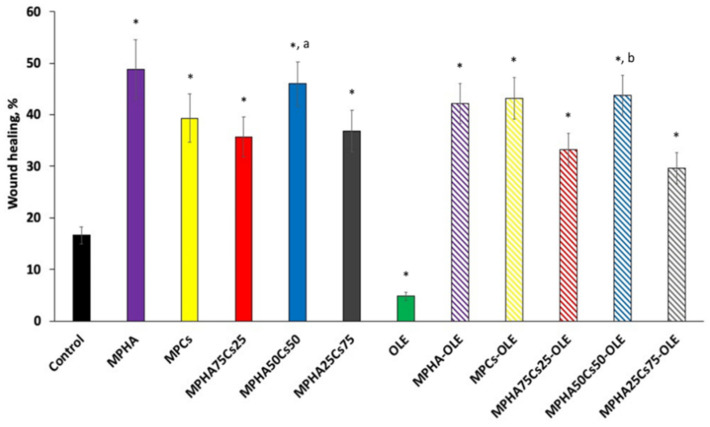
Wound closure rates expressed as percentage of scratch closure after 24 h compared to initial area. Control consists in untreated cells. Means ± SD (*n* = 6). * *p* < 0.05 vs. control; ^a^ *p* < 0.5 vs. MPHA75Cs25 and MPHA25Cs75; ^b^ *p* < 0.5 vs. MPHA75Cs25-OLE and MPHA25Cs75-OLE.

Figure 9 shows the wound healing percentages for all the studied samples. From the data in Figure 9, it can be observed that OLE is not able to accelerate the healing of wounds, in fact, it slows it down, as a % healing was significantly lower than the control. This data appears in contrast to other literature studies in which OLE was found to be active both as an antibacterial and an accelerator of wound healing. The concentrations tested were higher than that applied in the present work [37]. In another work, it was shown in vivo that wound healing was promoted by olive leaf extracts and that this effect was greater the greater was the scavenging ability of the extract [38]. This activity has been attributed to the presence of oleuropein in the extract which was also the main component of our extract. However, it must be borne in mind that in the experimental conditions of in vitro tests showed that oleuropein could rapidly be oxidized and the oxidized product would exert no action on wound healing. Furthermore, from the data in Figure 9 it can be hypothesized that the oxidized form of oleuropein could be responsible for the worsening of wound healing compared to the untreated control. Instead, MPHA and MPCs were found to be very effective in promoting wound healing and the differences were not significant. Likewise, no significant differences were observed between medicated and non-medicated MP.

**Figure 10:**
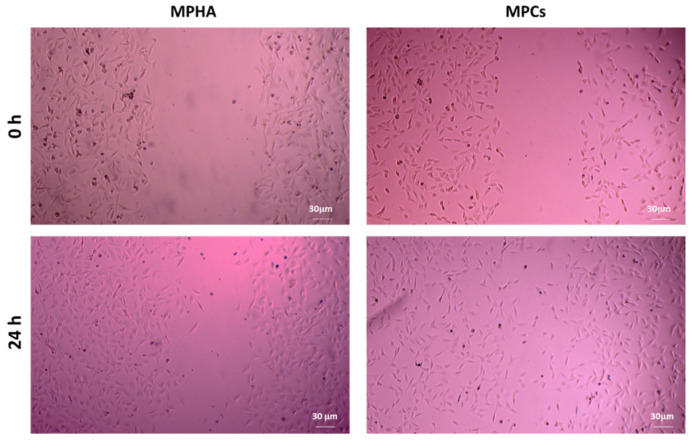
Representative micrographs (4× magnification) of BALB/3T3 clone A31 murine embryonic fibroblast cell monolayers treated with MPHA (**left**) or with MPCs (**right**).

From a careful observation of the photomicrographs shown in Figure 10, it is possible to observe how BALB/3T3 clone A31 cells treated with MPHA promote cell proliferation. It is in fact known how HA acts in the first phase of wound healing, that is, in the proliferative phase [39]. The qualitative analysis of the images shown in Figure 10 also allowed noting how the presence of MPCs rather favors cell migration. In fact, Cs, by mimicking the glycosaminoglycans portion of the extracellular matrix, allows cells to activate the mechanisms of cell migration, which represents the second phase of healing. The qualitative analysis of the images shown in Figure 10 also allowed noting how the presence of MPCs rather favors cell migration. In fact, Cs, by mimicking the glycosaminoglycans portion of the extracellular matrix, allows cells to activate the mechanisms of cell migration, which represents the second phase of healing [14,40].

This interesting observation prompted us to evaluate the opportunity to create a device capable of promoting cell proliferation in the wound site thanks to the presence of HA and simultaneous cell migration thanks to the presence of Cs [11]. Furthermore, the dual function of Cs would favor both cell migration, but also an important antimicrobial action [14,40], the latter strengthened by the OLE [41] which had previously been shown to have anti-inflammatory activity [19]. In Figure 9, it is observed that the MPHA50MPCs50 mixture—medicated or not—has a significantly higher wound healing activity than all the other mixtures tested, probably because the presence of the two components must be balanced to achieve more effective wound healing.

## 4. Conclusions

The OLE-loaded HA and Cs-based MP studied in this work were shown to have characteristics suitable for making a spray patch, thus giving a second life to the waste products of olive growers. The particles of the spray patches were less than 5 µm of size, were able to swell by absorbing the wound exudate water, were able to slowly release OLE and protect it from degradation, and were able to accelerate wound healing. A mix containing 50% MPHA-OLE and 50% MPCs-OLE, although shown by in vitro scratch studies to be unable to significantly improve the performance of unmixed HA- or Cs-based MP, nevertheless, it turned out to be very interesting. Firstly the MPCs, unlike the MPHA, were able to control the release of OLE and to protect it from degradation; secondly, it was observed from the photomicrographs that the MPCs can promote the migration of fibroblasts, whereas the MPHA can promote their proliferation. However, in vivo studies will be necessary before a possible synergistic effect of the two MP types can be stated. In such studies, the microparticles are intended for application on the wound as a spray.

## Data Availability

Not applicable.
